# Comparison of bacterial vaginosis symptoms reported in social media vs. those reported by patients

**DOI:** 10.3389/frph.2025.1549331

**Published:** 2025-02-28

**Authors:** Andriana H. Velmahos, Briah Cooley Demidkina, Caroline M. Mitchell

**Affiliations:** ^1^ Boston University Chobanian and Avedisian School of Medicine, Boston, MA, United States; ^2^ Vincent Center for Reproductive Biology, Massachusetts General Hospital, Boston, MA, United States; ^3^ Department of Obstetrics, Gynecology and Reproductive Biology, Harvard Medical School, Boston, MA, United States

**Keywords:** bacterial vaginosis, vaginal health, vaginal discharge, vulvovaginal itching, social media

## Abstract

**Introduction:**

There is an increasing tendency to seek health information online rather than through medical professionals. However, the easy accessibility of information online allows for an increased risk of encountering medical misinformation.

**Methods:**

We compared descriptions of symptomatology of bacterial vaginosis (BV) on four social media platforms: Instagram, Facebook, YouTube and X (Twitter). We then compared the frequency of discussion of symptoms with patients' report of symptoms in clinically diagnosed BV, vulvovaginal candidiasis and vulvodynia.

**Results:**

Social media was more likely to report burning (45% of posts), itching (45%) or pain (23%) as symptoms of BV than patients (moderate-severe itching 30%, burning 21% or pain 21%). Although pain was reported more often by people with vulvodynia, other symptoms were not different across diagnoses.

**Discussion:**

Social media overemphasizes the ability of symptoms to allow self-diagnosis of vaginitis, which can lead to delayed treatment, highlighting the need to promote accurate health information online.

## Introduction

Bacterial vaginosis (BV) is the most prevalent vaginal infection among women of reproductive age, impacting approximately 29% of women in the United States (1, 2). The cause of vaginal symptoms cannot be inferred simply from type of symptoms, or appearance of discharge (3, 4). However, in the absence of consistent access to clinical care, many people attempt to self-diagnose and manage vaginal discharge symptoms themselves (5).

There is an increasing tendency to seek health information online rather than through medical professionals. The internet can be a critical resource which allows for easily accessible information about conditions that carry social stigma, such as vaginal health and BV. However, the easy accessibility of information online allows for an increased risk of encountering medical misinformation, which can misguide individuals making their own healthcare decisions (6).

We hypothesized that online discourse may overemphasize associations between BV and symptoms such as pain, burning and irritation, which can occur with many different clinical conditions. By investigating how online discussions of these symptoms, as well as discharge, odor and itching, and comparing this with clinical data, we aimed to identify how well the public perception matches the clinical reality of BV symptoms and promote the dissemination of accurate, accessible medical information in the digital age.

## Methods

### Data collection and sampling: social media

In this descriptive study, we explored posts about symptoms associated with bacterial vaginosis (BV) on Instagram, Facebook, X (formerly Twitter), and YouTube. We used relevant hashtags and keyword searches to identify posts. To identify relevant hashtags on Instagram, we used the platform's search function by entering “#bacterialvaginosis.” Instagram then generated a list of “hashtag recommendations” (similar to the way that Google does “search suggestions”). The hashtag recommendations on Instagram are presented in descending order based on the number of posts associated with each. We selected the top three hashtags with the highest post counts from this list: #bacterialvaginosis, #bacterialvaginosistreatment, and #bacterialvaginosisawareness. We then carried over these same hashtags to Facebook and Twitter. We limited our inclusion to the first 30 posts mentioning BV symptomatology under each hashtag for each platform. In cases where we reviewed all posts before reaching 30 relevant posts for a hashtag, we included all available relevant posts. For YouTube, we queried “Bacterial Vaginosis” and selected the first 30 videos mentioning BV symptomatology. In general, posts did not have to be exclusively focused on BV symptoms; rather, they simply needed to reference them within the broader discussion. Only posts that clearly attributed symptoms to BV were counted for this analysis. Posts mentioning multiple diagnoses were also included, but these specifically compared conditions and clearly described which symptoms were associated with each. This approach enabled the inclusion of posts addressing various aspects of BV, such as general awareness, comparisons to other vaginal infections, and discussions about management strategies.

We excluded duplicates, posts uploaded before 2014, non-English posts, and posts with fewer than 5 interactions –defined as likes, comments, or views. Due to the dynamic nature and personalization of individual social media accounts, all data collection was completed within one week (August 5–9, 2024) by a single researcher. This approach yielded 112 eligible posts. Each post was reviewed to determine its number of interactions and the symptoms it mentioned in correlation to BV.

We recorded the frequency of posts reporting symptoms of discharge, odor, itching, irritation, pain, or burning associated with BV. Terms such as irritation, dryness, redness, and inflammation were grouped under the category of “irritation.” If information was present in the post, we further classified pain and burning as occurring during urination or intercourse. Irritation was defined to include any reports of irritation, discomfort, inflammation, dryness, or redness.

### Data collection and sampling: clinical population

To evaluate clinical reporting of symptoms in a patient population, we conducted a retrospective review of symptoms reported by patients enrolled in an observational cohort sample of women over 21, non-pregnant, presenting to the Massachusetts General Hospital (MGH) Gynecology or Vulvovaginal Disorders Clinic between August 2014 and August 2017 (7). The protocol was approved by the Massachusetts General Hospital Human Subjects Committee (2014P001066). All participants signed informed consent, completed a symptom severity questionnaire, and provided a vaginal swab for Nugent scoring.

For this analysis, we included all participants presenting for annual exam, or who were diagnosed with BV, vulvodynia, or a yeast infection. We excluded participants with vulvar dermatoses, menopause, HSV, DIV, idiopathic symptoms, or multiple diagnoses. Diagnosis of BV was either by the clinician seeing the patient or by the research team using a gram-stained vaginal fluid Nugent score of 7–10. Diagnosis of yeast infection was by fungal culture. Diagnosis of vulvodynia was made by the clinician, using the definition of vulvovaginal discomfort lasting longer than 3 months without an obvious alternative cause. All participants had a vaginal fluid Gram stain scored according to Nugent criteria.

The symptom questionnaire asked about the presence of the following symptoms in the past 4 weeks: vulvar itch, vulvar burning, vulvar pain, vaginal itch, vaginal burning, vaginal pain, vaginal discharge, vaginal odor. Each was scored on a 6-point scale: none, mild, mild-moderate, moderate, moderate-severe, severe. For this analysis we used the highest reported score of either vulvar or vaginal symptoms, and grouped people as None, Mild (which included mild and mild-moderate) or Moderate-Severe (which included Moderate, Moderate-Severe and Severe). We chose to group moderate-severe symptoms and focus on these based on the assumption that for a symptom to be mentioned in a social media post it needed to be sufficiently bothersome.

### Statistical analysis

We used chi-square to compare the proportion of participants who reported vulvovaginal burning, pain, or itching between social media vs. clinical cohort, across clinical diagnoses within the cohort.

## Results

**Figure 1:**
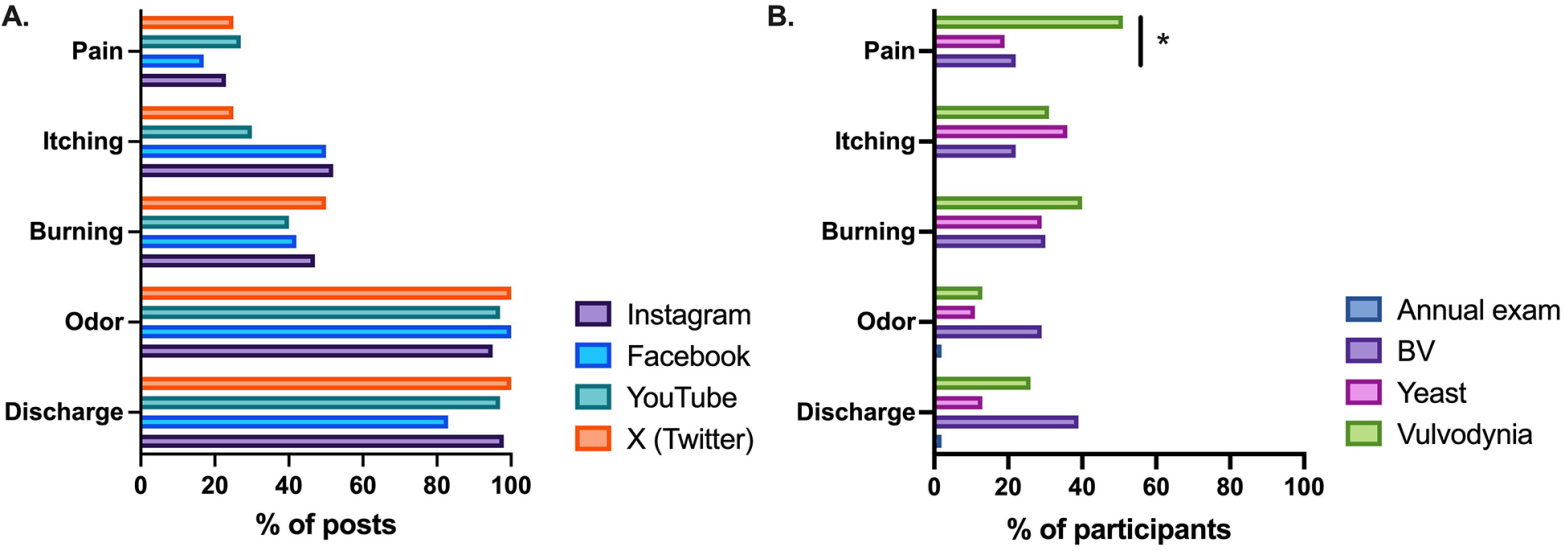
**(A)** Social media platforms frequently report discharge, odor, itching, burning and pain as symptoms of bacterial vaginosis (BV). **(B)** People presenting for annual exam had few symptoms, but there were few differences in report of moderate-severe symptoms between people with BV vs. yeast vs. vulvodynia (*p < 0.05 for comparison excluding annual exam).

On the social media platforms Instagram, Facebook, YouTube, and X (formerly Twitter), vaginal discharge (108/112, 96%)) and malodor (109/112, 97%)) were the two most frequently reported symptoms (Figure 1A). Itching (50/112, 45%) and burning (50/112, 45%) were mentioned in almost half of all social media posts, while pain was reported in association with BV in 23% (26/112) of all social media posts (Supplementary Table 1). YouTube was the platform most likely to have posts on BV that also reported pain (8/30, 27%). Most posts mentioning burning associated it with urination (38/50, 76%). YouTube was the sole social media platform that mentioned burning with intercourse, being reported in 17% of YouTube posts discussing burning (2/12). Among posts mentioning pain, pain with urination (10/26, 38%) was reported twice as often as pain with intercourse (5/26, 19%) (Supplementary Table 2).

A total of 140 cohort participant had questionnaire data available (Table 1). Among the 23 people with BV, the most commonly reported moderate-severe symptom was discharge (39%), followed by burning (30%) and odor (29%) (Figure 1B). When including any report of symptoms (mild-severe) the most commonly reported symptoms were discharge (70%) and odor (57%), followed by itching (52%) (Supplementary Table 3).

**Table 1:** Baseline characteristics of participants in the observational cohort. Participants are premenopausal people, attending their first visit at the MGH Vulvovaginal Disorders Clinic or presenting for an annual exam in the Gynecology clinic.

Characteristic	Annual	BV	Yeast	Vulvodynia
*N*	51	23	31	35
Age	37 ± 8	34 ± 10	32 ± 7	31 ± 6
Race				
Asian	4 (8%)	0 (0%)	1 (3%)	3 (9%)
Black	0 (0%)	1 (4%)	2 (6%)	3 (9%)
White	46 (90%)	22 (96%)	28 (90%)	28 (80%)
Non-specified	1 (2%)	0 (0%)	0 (0%)	1 (3%)
Ethnicity				
Hispanic	3 (6%)	2 (91%)	2 (6%)	1 (3%)
Non-Hispanic	48 (94%)	21 (9%)	29 (94%)	34 (97%)
Contraception				
None	17 (37%)	8 (44%)	7 (27%)	15 (50%)
OCP	16 (35%)	5 (28%)	9 (35%)	8 (27%)
IUD	5 (11%)	3 (17%)	5 (19%)	2 (7%)
NuvaRing	2 (4%)	0	2 (8%)	2 (7%)
DMPA/Nexplanon	1 (2%)	1 (6%)	1 (4%)	1 (3%)
Sterilization	4 (9%)	1 (6%)	2 (8%)	2 (7%)

When comparing the frequency of symptom report between the social media platforms and cohort participants, itch, discharge and odor were significantly less often reported as moderate-severe by participants than the frequency of their mention in social media posts: discharge 39% vs. 96% (*p* < 0.001), odor 29% vs. 96% (*p* < 0.001), itch 22% vs. 45% (*p* = 0.04) (Supplementary Tables 1 and 3). Pain and burning were reported with similar frequency: pain 22% vs. 20% (*p* = 0.88), burning 30% vs. 45% (*p* = 0.19).

To assess the reliability of symptoms for identifying BV, we compared participants who came for an annual exam or were diagnosed with BV, yeast, or vulvodynia (Figure 1B, Supplementary Table 3). In patients presenting for an annual exam, most reported no discharge (61%), no odor (75%), no burning (100.0%), no itching (98.0%), and no pain (98.0%). When we compared report of moderate to severe symptoms between patients with BV vs. yeast vs. vulvodynia the only symptom with a statistically significant difference was moderate-severe pain: 51.4% vulvodynia vs. 21.7% BV vs. 19.4% yeast (*p* = 0.007).

## Discussion

This study compared the description of BV symptomatology in online discourse compared to what is reported by patients with a confirmed diagnosis. We included symptoms frequently discussed online –such as vulvovaginal burning, pain, and itching– that are not part of the classic clinical description of BV. Additionally, this study compared these symptoms across various vaginal disorders to determine whether these vaginal symptoms could effectively distinguish between conditions in a clinical setting. Our findings show high search volumes on topics related to BV, highlighting patients' increased tendency to seek answers and information online. However, our findings revealed that online discourse aimed at helping patients recognize BV often emphasize symptoms that are not exclusively indicative of BV.

Analysis of social media platforms demonstrated that BV is reported to be associated with itching and burning, particularly burning with urination, approximately half of the time. Pain was mentioned less frequently in online discourse but still reported a quarter of the time. In contrast, our clinical population diagnosed with BV reported burning, pain, and itching less than a quarter of the time. In addition, burning and itching were present and equally prevalent in patients with yeast infections or vulvodynia, and pain was more prevalent in vulvodynia over yeast and BV. Discharge and odor were mentioned in nearly all social media posts. In our clinical population, these symptoms were notably more common in patients with BV compared to those with other vulvovaginal disorders, yet only one-third of patients diagnosed with BV reported either of these symptoms.

Our findings align with previous research showing that individuals often turn to the internet for health information, especially for conditions with social stigma, such as sexual and gynecologic health (8, 9). However, vaginal symptoms in the absence of lab tests poorly distinguish between causes of vaginitis (10, 11). Our findings emphasize the extent to which online discussions may mislead patients by suggesting that symptoms can accurately allow self-diagnosis. Unfortunately, BV is often under- or mis-diagnosed in the clinic, due to lack of appropriate testing and evaluation (10). The high prevalence of internet use for health-related decisions highlights the need to promote clear, accessible, and evidence-based clinical guidelines to mitigate the complications of unmanaged diseases.

A strength of this study was its analysis of four widely used social media platforms –YouTube, Facebook, Instagram and X (formerly Twitter)– with the most common hashtag terms for BV to provide a snapshot of the online discourse surrounding BV symptomatology. Our clinical cohort included both people presenting for clinical complaints as well as people who were simply presenting for annual exam and had clear diagnostic criteria for BV and yeast. However, limitations include that the social media data were collected by one individual over a brief period and is subject to the ever-changing nature and personalization of online content. We did not include TikTok in our review of social media sites, which is increasingly a trusted source of information for people. The questionnaire data was collected between 7 and 10 years prior to the social media assessment, and so common terminology may be different between the two time periods. Additionally, the cohort does not fully represent the diversity of the broader population due to the primarily White race of participants and smaller sample size, limiting the generalizability of our findings.

This study highlighted discrepancies between online discourse and clinical evidence regarding the symptoms of BV. In comparison to our clinical population, social media disproportionately characterized burning and itching as being characteristic of BV. In addition, our clinical population demonstrated that symptoms of burning, itching, and pain are present across many vulvovaginal disorders, and are not sufficient to distinguish between BV and other conditions. These discrepancies in online discourse and clinical practice underline the need for more accurate online information to ensure increased recognition and better health outcomes for patients experiencing vaginal symptoms.

## Data Availability

The datasets presented in this article are not readily available because Use of the data would require review and approval by an appropriate human subjects committee. Requests to access the datasets should be directed to caroline.mitchell@mgh.harvard.edu.
