# Supplementary material for: Comparison of bacterial vaginosis symptoms reported in social media vs. those reported by patients
Source: Front Reprod Health. 2025 Feb 28;7:1549331. doi: 10.3389/frph.2025.1549331 (PMC11906998; doi:10.3389/frph.2025.1549331)
Supplement: Supplementary file 1 [file Table1.docx]

**SUPPLEMENTAL MATERIAL**

Comparison of bacterial vaginosis symptoms reported in social media vs. those reported by patients

Andriana H. Velmahos^1^, Briah Cooley Demidkina^2^, Caroline M Mitchell^2,3^

^1^ Boston University Chobanian and Avedisian School of Medicine, Boston, MA

^2^ Vincent Center for Reproductive Biology, Massachusetts General Hospital, Boston, MA

^3^ Harvard Medical School, Boston, MA

Supplementary Table 1: Number of Social Media Posts Mentioning BV Symptoms

| **Symptoms** |  | **Total**  **(N=112)** | **Instagram**  **(N=66)** | **Facebook**  **(N=12)** | **YouTube**  **(N=30)** | **X (Twitter)**  **(N=4)** |
| --- | --- | --- | --- | --- | --- | --- |
|  | **Burning (Total)** | 50 | 31 | 5 | 12 | 2 |
|  | **Discharge** | 108 | 65 | 10 | 29 | 4 |
|  | **Malodor** | 109 | 63 | 12 | 30 | 4 |
|  | **Asymptomatic** | 31 | 11 | 2 | 18 | 0 |
|  | **Itching** | 50 | 34 | 6 | 9 | 1 |
|  | **Irritation** | 21 | 16 | 1 | 3 | 1 |
|  | **Pain (Total)** | 26 | 15 | 2 | 8 | 1 |

Supplementary Table 2. Percent of Social Media Posts that Mentioning Burning or Pain Which Specify Pain in Association with Urination or Intercourse

|  |  | **Total** | **Instagram** | **Facebook** | **YouTube** | **X (Twitter)** |
| --- | --- | --- | --- | --- | --- | --- |
| **Symptoms** | **Burning w/ urination** | 76 | 84 | 40 | 67 | 100 |
|  | **Burning w/ intercourse** | 4 | 0 | 0 | 17 | 0 |
|  | **Pain w/ urination** | 38 | 27 | 50 | 63 | 0 |
|  | **Pain w/ intercourse** | 19 | 7 | 50 | 38 | 0 |

Supplementary Table 3. Symptom Severity in Participants Presenting for an Annual Exam or with BV, Yeast, or Vulvodynia.

|  | | | **% OF PARTICIPANTS REPORTING (n)** | | | |  |
| --- | --- | --- | --- | --- | --- | --- | --- |
| **SYMPTOM** |  | **SEVERITY** | **Annual**  **(N = 51)** | **BV**  **(N = 23)** | **Yeast**  **(N = 31)** | **Vulvodynia**  **(N = 35)** | **p-value**  **p-value*** |
|  | **Burning** | **None** | 100.0 (51) | 52.2 (12) | 48.4 (15) | 25.7 (9) | <0.001  0.24* |
|  |  | **Mild** | 0.0 (0) | 17.4 (4) | 22.6 (7) | 34.3 (12) |  |
|  |  | **Mod-Severe** | 0.0 (0) | 30.4 (7) | 29.0 (9) | 40.0 (14) |  |
|  | **Itching** | **None** | 98.0 (50) | 47.8 (11) | 41.9 (13) | 31.4 (11) | <0.001  0.55* |
|  |  | **Mild** | 2.0 (1) | 30.4 (7) | 22.6 (7) | 37.1 (13) |  |
|  |  | **Moderate-Severe** | 0.0 (0) | 21.7 (5) | 35.5 (11) | 31.4 (11) |  |
|  | **Pain** | **None** | 98.0 (50) | 56.5 (13) | 51.6 (16) | 17.1 (6) | <0.001  0.007* |
|  |  | **Mild** | 2.0 (1) | 21.7 (5) | 29.0 (9) | 31.4 (11) |  |
|  |  | **Moderate-Severe** | 0.0 (0) | 21.7 (5) | 19.4 (6) | 51.4 (18) |  |
|  | **Discharge** | **None** | 60.8 (31) | 30.4 (7) | 41.9 (13) | 42.9 (15) | 0.002  0.24* |
|  |  | **Mild** | 37.3 (19) | 30.4 (7) | 45.2 (14) | 31.4 (11) |  |
|  |  | **Moderate-Severe** | 2.0 (1) | 39.1 (9) | 12.9 (4) | 25.7 (9) |  |
|  | **Odor^+^** | **None** | 74.5 (35) | 42.9 (9) | 46.4 (13) | 56.3 (18) | 0.019  0.38* |
|  |  | **Mild** | 23.4 (11) | 28.6 (6) | 42.9 (12) | 31.3 (10) |  |
|  |  | **Moderate-Severe** | 2.1 (1) | 28.6 (6) | 10.7 (3) | 12.5 (4) |  |
| *Chi-square test excluding annual exams  ^+^N for Odor were 47, 21, 28, 32 respectively due to missing data from participants reporting their symptoms. | | | | | | | |
